# Editorial: Innovations in development, translational research and manufacturing of CAR T cells

**DOI:** 10.3389/fimmu.2024.1504906

**Published:** 2026-03-26

**Authors:** D Krones, U Koehl, H Negre, Q Rafiq, S Goldrick, M Hudecek

**Affiliations:** ^1^ Department of Internal Medicine II, Chair of Cellular Immunotherapy, University Hospital Würzburg, Würzburg, Germany; ^2^ Fraunhofer Institute for Cell Therapy and Immunology (IZI) and Branch Site Würzburg, Leipzig, Germany; ^3^ on behalf of EU IMI-2 Innovative Medicines Initiative T2EVOLVE – Accelerating Development and Improving Access to CAR and TCR-engineered T Cell Therapy in Europe, Würzburg, Germany; ^4^ on behalf of EU IMI Innovative Medicines Initiative imSAVAR – Immune Safety Avatar: Nonclinical Mimicking of the Immune System Effects of Immunomodulatory Therapies, Leipzig, Germany; ^5^ Instiute for Clinical Immunology, University of Leipzig, Leipzig, Germany; ^6^ Fraunhofer Cluster of Excellence for Immune-mediated Disease, Leipzig, Germany; ^7^ Cluster4Future SaxoCell, Leipzig, Germany; ^8^ CELLforCURE by Seqens, Les Ulis, France; ^9^ University College London, Department of Biochemical Engineering, S2.08 (Bernard Katz Building) University College London, London, United Kingdom; ^10^ on behalf of Collaborative Research Center Transregio 338 LETSimmun – Lymphocyte Engineering for Therapeutic Synthetic Immunity, Leipzig, Germany

**Keywords:** cell therapy, immune therapy, oncology, haematology, CAR T cell therapy, CAR NK cell therapy

The surging development of cellular immunotherapies sparked the creation of this Research Topic dedicated to Innovations in Development, Translational Research and Manufacturing of CAR T cells. This Research Topic addresses the current benefits observed with CAR T-cell immunotherapy, which raises the question of scalability of CAR T-cell products to provide access to a wide range of patients. Current improvements include both automation combined with artificial intelligence (AI) as well as non-viral gene transfer to improve safety, lower complexity and costs. In addition, data-driven knowledge about various diseases is changing the field, moving from hematological to oncology patients as well as to autoimmune diseases.

The clinical translation and correlative studies of CAR-T cell therapy continues to evolve through studies that refine our understanding of treatment dynamics, patient outcomes, and mechanisms underlying therapeutic success. A key contribution in this area comes from the work of García-Calderón et al., who performed a detailed kinetic and immunophenotypic analysis of circulating CD19 CAR-T cells in B-cell malignancies. By validating optimized flow-cytometric and digital PCR monitoring methods and linking early CAR-T expansion profiles to toxicities such as CRS and ICANS, the study correlates robust tools for real-time clinical monitoring. Importantly, their data identify PD-1/LAG-3 co-expression and reduced CD107a levels at peak expansion as early biomarkers associated with improved long-term disease control, underscoring the importance of *in-vivo* CAR-T cell surveillance in guiding clinical decision-making.

Complementary insights are offered by Odak et al., who applied a comprehensive 31-color spectral flow cytometry approach to evaluate CAR-T cell phenotype stability over six months. Their findings demonstrate largely stable expression of activation and exhaustion markers *in vivo*, while unsupervised clustering of infusion products identified distinct immune signatures associated with development of ICANS. This study strengthens the link between infusion-product phenotypes and clinical toxicity, emphasizing the impact of pre-infusion immune profiling.

Further broadening translational perspectives, Karsten et al. present an extensive clinical review of CAR-T cell applications across hematologic malignancies and beyond current indications. Their analysis of disease-specific challenges such as antigen heterogeneity, fratricide in T-cell malignancies, and tumor microenvironment as well as induced immunosuppression highlights the need for tailored CAR-T designs and patient-specific therapeutic strategies. The review underscores how clinical translation in new disease entities depends on integrating mechanistic insights and next-generation engineering approaches.

Ammar et al. examine the regulatory landscape governing early-phase evaluation of multiple CAR-T product versions in the EU. By describing how iterative and adaptive development strategies such as umbrella trials and structured assessment of closely related CAR constructs can be more efficiently integrated within current regulatory frameworks, this work addresses a crucial dimension of translation: the pathway that enables laboratory innovations to reach patients within a safe, flexible, and accelerated clinical development process are key insights delivered by the T2EVOLVE consortium.

Innovation in CAR-T cell technology is propelled by new engineering strategies, improved gene-delivery technologies, and refined tools to evaluate and control engineered immune cells. Among the contributions in this Research Topic, the study by Svec et al. represents a conceptual advance in CAR-T safety engineering. The authors introduce a CAR-based cellular safeguard system capable of selectively depleting therapeutic CAR-T cells *in vivo*, using a secondary CAR (“anti-CAR CAR”) that recognizes a peptide tag incorporated into the CAR construct. Their work demonstrates potent, antigen-specific elimination of functional CAR-T cells *in vitro* and in multiple mouse models, including the reversal of long-term B-cell aplasia — a key on-target/off-tumor toxicity in CD19 CAR-T therapy. The ability to precisely remove engineered T cells without conditioning regimens highlights a highly modular and clinically relevant safety switch, expanding the toolbox for controllable CAR-T therapies and informing next-generation designs aimed at minimizing late toxicities and enhancing clinical manageability.

Complementary technological advancements further broaden the landscape of CAR-T development. Kapitza et al. introduce a CD62L-targeted lentiviral vector capable of preferentially transducing less-differentiated T-cell subsets, which is a phenotype correlated with superior persistence and antitumor activity. The ability to enrich for early-memory CAR-T cells during manufacturing, without additional sorting steps, represents a powerful innovation that not only streamlines production and improves the functional quality of the final cell product, but rather opens possibilities for *in vivo* gene transfer.

In parallel, Rauch-Wirth et al. present optimized peptide nanofibrils that significantly enhance viral transduction efficiency in T and NK cells. These low-immunogenicity, aggregation-resistant peptides offer an accessible and cost-effective solution for improving gene-delivery kinetics, with direct relevance for large-scale CAR-T and CAR-NK manufacturing workflows.

Beyond engineering innovations, efforts to improve standardization and functional evaluation of CAR constructs are exemplified by Wang et al., who developed a versatile synthetic bead-based platform for CAR-T activation and characterization. This system enables controlled engagement of CARs and co-stimulatory receptors, eliciting activation, cytokine secretion, degranulation, and proliferation comparable to conventional target-cell assays. The resulting improvements in reproducibility and scalability address a longstanding need for standardized functional testing across CAR-T development pipelines.

Other studies highlight innovative biological concepts shaping next-generation CAR therapies. Calviño et al. explore the generation of universal allogeneic CAR-T cells for AML using combined CRISPR editing and Sleeping Beauty transposon delivery. Their HLA-I/TCR-knockout CD33-CAR-T cells maintain robust *in vitro* and *in vivo* antitumor activity, offering a feasible platform for off-the-shelf CAR-T products with reduced risk of GVHD and simplified manufacturing requirements.

Meanwhile, Staudt et al. detail emerging links between the microbiome and engineered T-cell immunity, highlighting how commensal metabolites and microbial community states shape T-cell metabolism, persistence, and resistance to exhaustion representing an increasingly important area for therapeutic modulation and patient stratification.

Expanding beyond T cells, Ciulean et al. demonstrate the potential of CAR-NK cells targeting CD44v6 for head and neck squamous cell carcinoma, optimizing viral pseudotyping strategies to enhance NK-cell transduction and showing substantially increased cytotoxicity against solid tumor cell lines. Their findings reinforce the role of CAR-NK cells as a complementary or alternative platform to CAR-T therapy, especially in settings where allogeneic use and safety are critical considerations

The manufacturing of CAR-T cells remains one of the most decisive determinants of product quality, clinical performance, and scalability. Innovations across process optimization, automation, and data-driven control are reshaping how advanced cellular products are produced. Within this context, the study by Aleksandrova et al. provide a comprehensive contribution by presenting a fully automated, GMP-compliant workflow for clinical-grade CD20 CAR-T cells manufactured on the CliniMACS Prodigy^®^ platform for patients with stage III/IV melanoma. The authors demonstrate highly consistent product composition across two independent production sites, with high T-cell purity, defined CD4/CD8 ratios, and significant transduction efficiencies by day 12. Their dataset further shows robust median expansion, generation of predominantly central memory CAR-T cells, and preserved functional activity as evidenced by CAR-induced cytokine secretion and antigen-dependent proliferation. This work underscores the translational importance of decentralized, point-of-care manufacturing, enabling harmonized product quality and reliable scalability which represent core challenges for broadening access to CAR-T therapies.

Perspectives on strengthening manufacturing robustness are offered by Colina et al., who empahsize the biological and technological variables that influence CAR-T cell expansion, phenotype, and functional fitness. Their analysis integrates experimental and computational approaches, including single-cell technologies, metabolic modulation, cytokine engineering, and in silico modeling, to map how media composition, activation strategies, gene-delivery methods, and culture duration influence key quality attributes. The review argues for a convergent future where computational modeling and experimental perturbation are used together to predict manufacturing outcomes and guide decisions earlier in the process.

Process optimization is examined in depth by Hood et al., examine process optimization by application of an in-depth quality-by-design (QbD) framework to systematically analyze critical process parameters such as seed-train duration, number of activation steps, initial seeding density, and IL-2 concentration. Through design-of-experiments screening followed by validation in automated stirred-tank bioreactors, they demonstrate that modifications such as reducing activation steps and shortening seed-train time markedly improve CAR-T yield, metabolic efficiency, and exhaustion-marker profiles. Importantly, the optimized parameters translate across donors and remain robust during scale-up, illustrating the power of QbD to create data-driven, transferable manufacturing protocols suited for industrial adoption.

A broader transformation in the field emerges from the perspective by Bäckel et al., who explore how Artificial Intelligence (AI) can support the digitalization and automation of CAR-T bioprocessing. By mapping each manufacturing step to potential AI applications from descriptive analytics to prescriptive control, the authors highlight how automated production platforms generate rich datasets that can feed machine-learning models. These models could enable real-time prediction of expansion dynamics, optimization of harvest timing, detection of process deviations, and long-term cost reduction. The article positions AI as a key enabler for future smart manufacturing ecosystems, ultimately contributing to more reproducible, affordable, and scalable CAR-T production pipeline, thus seamlessly connecting to projects such as the EU-funded AIDPATH project.

In closing, we extend our sincere appreciation to the authors who have contributed their high-quality scientific work to this Research Topic. Their research and insights have been helpful in advancing the field of cellular immunotherapy. We would also like to thank our project partners from the EU IMI-2 Innovative Medicines Initiative, T2EVOLVE, and imSAVAR, EU HORIZON 2020 Project AIDPATH, Clusters4Future Project SaxoCell, TRANSCAN-3 Project SmartCAR-T as well as the Collaborative Research Center Transregio 338 LETSimmun, for their cooperation and support. The publications curated here were made possible through their dedication and shared commitment to supporting progress in immunotherapy research.

